# Meta-Regression Analyses to Explain Statistical Heterogeneity in a Systematic Review of Strategies for Guideline Implementation in Primary Health Care

**DOI:** 10.1371/journal.pone.0110619

**Published:** 2014-10-24

**Authors:** Susanne Unverzagt, Frank Peinemann, Matthias Oemler, Kristin Braun, Andreas Klement

**Affiliations:** 1 Institute of Medical Epidemiology, Biostatistics and Informatics, University Halle/Wittenberg, Halle (Saale), Germany; 2 Children's Hospital, University of Cologne, Cologne, Germany; 3 Section of General Practice, Institute of Medical Epidemiology, Biostatistics and Informatics, University Halle/Wittenberg, Halle (Saale), Germany; University of Glasgow, United Kingdom

## Abstract

This study is an in-depth-analysis to explain statistical heterogeneity in a systematic review of implementation strategies to improve guideline adherence of primary care physicians in the treatment of patients with cardiovascular diseases. The systematic review included randomized controlled trials from a systematic search in MEDLINE, EMBASE, CENTRAL, conference proceedings and registers of ongoing studies. Implementation strategies were shown to be effective with substantial heterogeneity of treatment effects across all investigated strategies. Primary aim of this study was to explain different effects of eligible trials and to identify methodological and clinical effect modifiers. Random effects meta-regression models were used to simultaneously assess the influence of multimodal implementation strategies and effect modifiers on physician adherence. Effect modifiers included the staff responsible for implementation, level of prevention and definition pf the primary outcome, unit of randomization, duration of follow-up and risk of bias. Six clinical and methodological factors were investigated as potential effect modifiers of the efficacy of different implementation strategies on guideline adherence in primary care practices on the basis of information from 75 eligible trials. Five effect modifiers were able to explain a substantial amount of statistical heterogeneity. Physician adherence was improved by 62% (95% confidence interval (95% CI) 29 to 104%) or 29% (95% CI 5 to 60%) in trials where other non-medical professionals or nurses were included in the implementation process. Improvement of physician adherence was more successful in primary and secondary prevention of cardiovascular diseases by around 30% (30%; 95% CI -2 to 71% and 31%; 95% CI 9 to 57%, respectively) compared to tertiary prevention. This study aimed to identify effect modifiers of implementation strategies on physician adherence. Especially the cooperation of different health professionals in primary care practices might increase efficacy and guideline implementation seems to be more difficult in tertiary prevention of cardiovascular diseases.

## Introduction

Cardiovascular diseases (CVDs) are major causes of morbidity and mortality across developed countries [1]. Various guidelines are published that provide information on health care in the prevention and treatment of CVD [2]–[5]. A time gap between publication and implementation in clinical practice may evolve. In a recent systematic review, we found that using implementation strategies (versus not using implementation strategies) improved the adherence of physicians to guidelines on primary care of patients with CVDs [6]. We also found a considerable variability of treatment effects among the two evaluated groups.

This heterogeneity may be an artifact of methodological factors [7]–[9]. These factors may include differences in study design features such as predefined primary outcomes, unit of randomization [10], duration of follow-up [11], and risk of bias. High risk of bias may change the magnitude and even the direction of treatment effects [12]–[16]. On the other hand, heterogenic effects of implementation strategies may result from a wide range of clinical factors such as variations in the patients with CVD in primary care, types and timing in the measurement of adherence, and special characteristics of investigated strategies [7]–[9], [17], [18]. Implementation strategies may be characterized as unimodal or multimodal or by their distinct quality improvement components (e.g. Shojania 2004 [19]). Strategies to improve physician adherence are complex interventions and an understanding of the effectiveness of these interventions is based on the assessment of what works best in different populations, circumstances and contexts [9], [20], [21]. These conditions may cause variations in treatment effects across different studies included in the review and provide an opportunity to identify clinical factors that may modify the treatment effect on physician adherence and increase scientific understanding [7]. Discordance between recommended and observed behaviour of physicians in the treatment of cardiovascular diseases is influenced by modifiable context-specific barriers as providers' and patients' knowledge of and attitudes towards adherence to health care recommendations and by external factors related to the health system including lack of policy support for chronic care and prevention or limited access to health-care resources [22].

Primary care frequently occurs in small health care organizations with a team of professionals consisting of physicians, nurses, and other professionals in the primary, secondary, and tertiary prevention of CVD with a variety of symptoms and other diseases. The differences among professionals and/or professions involved in the care of patients with different indications and severity of CVD may influence the quality of dissemination and implementation of guidelines [23].

Statistical heterogeneity describes the variability in treatment effect estimates between studies and may arise from methodological or clinical heterogeneity, from other unknown, unrecorded or unreported study characteristics, or may be due to chance [17]. The causes and the extent of heterogeneity should be evaluated as they may compromise the implications of systematic reviews [24]. Statistical heterogeneity between treatment effects estimated by individual studies can be visually assessed in forest plots, tested for statistical significance, and quantified using the percentage of total variation across studies I^2^ [25], [26], or the between-study variance τ^2^ [27], [28]. Heterogeneity may be explored by conducting subgroup analyses or weighted meta-regression in complex interventions [29], [30]. Meta-regression analysis can be used for a simultaneous exploration of potential methodological and clinical effect modifiers. One way of dealing with statistical heterogeneity in meta-analyses and meta-regression analyses is to incorporate a term to account for it in a random-effects model.

This study is a workup of a previously published systematic review [6]. The main aim is to explain the heterogeneity of findings from the original review to identify possible methodological and clinical heterogeneity factors that may have influenced estimated treatment effects of guideline implementation strategies on physician adherence in primary care of patients with CVD.

## Methods

### Study design

The design of this systematic review and the efficacy results of different implementation strategies in comparison to usual care have been previously published [6]. In brief, searches included Medline, Embase, the Cochrane Library, references of included studies, conference proceedings, register of ongoing trials and references of all included studies published between 1990 and 2012. The review considered all randomized studies that investigated guideline dissemination and their implementation into treatment of patients with CVD in primary care practices. Trials had to report guideline adherence of physicians over a minimum period of 3 months after initiating the implementation strategies. We extracted information on design, indication of patients according to the International Classification of Diseases-10 (ICD-10), implementation strategies and outcomes from all eligible trials into standardized data extraction tables. Implementation strategies were categorized as provider reminder systems, provider education, facilitated relay to data, audit and feedback, promotion of self-management, patient education, patient reminder, and organizational change [31]. The internal validity of trials was judged according to the Cochrane Collaboration risk of bias tool [10] with extension to cluster randomized trials (c-RCTs) [32]–[34] as high, unclear or low in six specific domains including bias in random sequence generation, allocation concealment, blinded outcome assessment, documentation of incomplete outcome data and selective reporting. Furthermore, baseline comparability between treatment groups and the use of adjustment methods to cope with potential imbalances in both cluster and individual characteristics were summarized as other sources of bias. All steps were done by two independent authors, disagreements were resolved by discussion until consensus was obtained. All treatment effect measures for the primary endpoint (physician adherence) were presented as odds ratios (OR) with 95% confidence intervals (CI) and recoded so that an OR higher than 1 indicates a beneficial effect with higher physician adherence in the experimental implementation strategy. Multiple endpoints were summarized using the mean of logarithmic ORs and ORs were recalculated from relative risks [10], standardized mean difference with standard deviation [35], and absolute frequencies of physician adherence in different groups. Results of c-RCTs without hierarchical modelling were corrected with the reported intra-cluster correlation coefficient and the mean number of patients per cluster [10]. Effect sizes were interpreted in categories of *small* to describe effect sizes ≤20%, *moderate* to describe effect sizes>20 and ≤50%, and *large* to describe effect sizes of >50% increase of physician adherence in comparison to passive implementation.

### Investigation of heterogeneity

In our first publication we explored treatment effects in subgroups of unimodal interventions, graphically displayed them in forest plots, and quantified the remaining heterogeneity of treatment effects using the I^2^ and τ^2^ values [6]. Due to the high heterogeneity between included trials we used the random-effects model for meta-analysis of the relevant comparisons of implementation strategies to usual care. This study adds a simultaneous assessment of the influence of multimodal implementation strategies and different effect modifiers on physician adherence in six random effects meta-regression models. We separately added six single sources of heterogeneity to binary variables describing the components of multimodal implementation strategies and investigated their influence on the treatment effect. Sources of heterogeneity included the staff responsible for implementation, level of prevention, definition of the primary outcome, unit of randomization, duration of follow-up and risk of bias domains.

We quantified the influence of all investigated sources of heterogeneity with ratios of odds ratios (ROR) comparing OR of studies with different values of the sources of heterogeneity (e.g. studies in secondary prevention with those in tertiary prevention of CVD). ROR with 95% confidence intervals (95%CI) not containing the null value (ROR = 1) will be interpreted as significant. Heterogeneity was measured using the τ^2^ statistics which estimates the between-trial variability. The amount of heterogeneity explained by different effect modifiers was described by the relative reduction of τ^2^. All modifiers were investigated as binary traits. Categorical traits (staff responsible for implementation, type of prevention) were recoded into dummy variables. We conducted the statistical analyses using RevMan5 for the systematic review and SAS 9.2 (PROC MIXED statement) for this study.

## Results

Meta-analyses of treatment effects on physician adherence revealed considerable heterogeneity in all subgroups of unimodal interventions [6]. In this study, altogether 75 trials were pooled in 84 comparisons between unimodal or multimodal active and passive implementation strategies (Table S1). Of these comparisons, 13 indicated a negative treatment effect with an OR<1, 17 a small effect size, 22 a moderate effect size, and 32 a large effect size of physician adherence to guidelines.

In the majority of trials the implementation strategies were directed at physicians (70 trials). Physicians were supported by nurses in 19 trials or other non-medical professionals such as pharmacists (12 trials), (study)-assistants (3 trials), health workers (2 trials), (peer-) supervisors, praxis managers, or other specialists (1 trial). In four trials, strategies were exclusively implemented by specialized nurses and in one trial by a team of nurses, Asian link (health-) workers, and community diabetes specialists. Most trials concentrated on patients in the secondary prevention of CVD, 10 trials additionally included patients in primary prevention, and 6 trials included patients in tertiary prevention. Only 6 trials were limited to primary prevention and 12 trials to tertiary prevention. The most successful strategies based on patient education and organizational change regularly included nurses (46 and 41% of trials) or other professionals (31 and 71% of trials) in the implementation process (Table 1).

**Table 1:** Investigated sources of heterogeneity in 75 trials.

Implementation strategies			Direction of implementation	Level of prevention	Design	Investigation of physician adherence as	Follow-up
Categories	Data source	Odds ratio; 95% CI	Physician/nurse/other professions	Primary/Secondary/Tertiary	RCT/c-RCT	Primary/secondary outcome	3–12 months/>12 months
Provider reminder systems	[49]–[70]	1.07; 0.93 to 1.23	22 (100%)/7 (32%)/5 (23%)	4 (18%)/18 (82%)/7 (32%)	3 (14%)/19 (86%)	17 (78%)/5 (22%)	20 (91%)/2 (9%)
Facilitated relay to data	[71]	2.01; 1.02–3.96	1 (100%)/0 (0%)/0 (0%)	0 (0%)/1 (100%)/0 (0%)	0 (0%)/1 (100%)	0 (0%)/1 (100%)	1 (100%)/0 (0%)
Audit and feedback	[55], [56], [59], [63], [72]–[79]	1.01; 0.73 to 1.40	12 (100%)/1 (8%)/3 (25%)	6 (50%)/9 (76%)/1 (8%)	2 (17%)/10 (83%)	7 (58%)/5 (42%)	9 (75%)/3 (25%)
Provider education	[49], [51], [59], [72], [73], [76], [78]–[96]	1.34; 1.08 to 1.65	23 (92%)/4 (16%)/4 (14%)	5 (20%)/15 (60%)/7 (28%)	1 (4%)/24 (96%)	16 (64%)/9 (36%)	19 (76%)/6 (24%)
Patient education	[55], [72], [80], [86], [96]–[105]	1.48; 1.08 to 2.01	11 (79%)/6 (46%)/4 (31%)	3 (23%)/10 (77%)/2 (14%)	5 (36%)/9 (64%)	3 (21%)/11 (79%)	10 (71%)/4 (29%)
Promotion of self-management	[71], [72], [83], [91], [97], [99], [102], [105]–[109]	1.08; 0.80 to 1.45	10 (83%)/4 (33%)/1 (8%)	1 (8%)/9 (75%)/3 (25%)	7 (58%)/5 (42%)	1 (8%)/11 (92%)	12 (100%)/0 (0%)
Patient reminders	[52], [97], [98], [101], [102], [104], [110]	0.81; 0.51 to 1.28	7 (100%)/4 (58%)/0 (0%)	0 (0%)/5 (83%)/2 (29%)	5 (71.4%)/2 (28.6%)	1 (14%)/6 (86%)	5 (71%)/2 (28%)
Organizational change	[69], [85], [93], [103], [111]–[123]	1.49; 1.21 to 1.82	15 (88%)/7 (41%)/12 (71%)	5 (29%)/14 (82%)/5 (29%)	8 (47%)/9 (53%)	6 (35%)/11 (65%)	13 (76%)/4 (24%)

Abbreviations: CI: confidence interval; c-RCT: cluster randomized controlled trial; RCT: randomized controlled trial.

Approximately half of the trials were pre-planned with physician adherence as the primary outcome (36 trials) and the other half of trials reported adherence as the secondary outcome or parameter describing the process of care (39 trials). Units of analyses were individual patients in 23 RCTs and practices in 52 c-RCTs. Implementation strategies that were directed to the staff of general practices, such as provider reminder systems, audit and feedback, provider education, and organizational change, were frequently investigated in c-RCTs with physician adherence as the primary outcome. On the other hand, trials on strategies directed to patients such as patient education, promotion of self-management, and patient reminders predominantly concentrated on patient-related outcomes and reported physician adherence to the implementation process mostly as a secondary outcome. Follow-up periods between 3 and 36 months were used to investigate the efficacy of the intervention with a median length of follow-up of 12 months. Of the 75 trials, 61 trials had follow-up periods of between 3 and 12 months and 14 had longer follow-up periods of up to 36 months (Table 1).

Of the 75 trials, 48 reported the method of randomization in the text. The treatment allocation of clusters or patients was described as concealed in 63 trials. Physician adherence was assessed on objective criteria (such as number of medications, or by external monitors) and/or blinded in 63 trials. In 61 trials, the analyses were done by intention-to-treat, both at the individual and at the cluster levels. Total numbers of dropouts were low (<10%) and their causes were given by group. Primary endpoints were pre-specified in sample-size calculations and were adequately reported in 47 trials. Other sources of bias were evident in 28 trials that made no use of appropriate methods for the adjustment of treatment effects on physician adherence to cope with potential imbalances in cluster and individual characteristics. Summarizing these results, only 15 trials had low risk of bias in all the investigated domains.

### Association between heterogeneity factors and estimated treatment effects

We calculated relative frequencies of negative, small, moderate, and large treatment effects depending on subgroups with special clinical and methodological characteristics. These characteristics were correlated with special implementation strategies, as shown in Table 2.

**Table 2:** Association between trial characteristics and treatment effect in 84 comparisons.

Potential effect modifier	Negative direction of effect	Small size	Moderate effect size	Large effect size
	OR<1.0	OR≥1.0 and OR≤1.2	OR>1.2 and OR≤1.5	OR>1.5
Implementation received by physician	12 (16%)	15 (19%)	21 (27%)	29 (38%)
Implementation received by nurse	4 (17%)	4 (17%)	6 (25%)	10 (42%)
Implementation received by other professionals	1 (4%)	6 (21%)	5 (18%)	16 (57%)
Primary prevention	2 (9%)	5 (24%)	4 (19%)	10 (48%)
Secondary prevention	11 (18%)	9 (15%)	17 (27%)	25 (40%)
Tertiary prevention	3 (14%)	7 (33%)	6 (29%)	5 (24%)
Design: c-RCT	10 (17%)	15 (25%)	14 (23%)	21 (35%)
Design: RCT	3 (12%)	2 (8%)	8 (33%)	11 (46%)
Adherence as primary outcome	5 (12%)	7 (17%)	13 (32%)	16 (39%)
Adherence as secondary outcome	8 (19%)	10 (23%)	9 (21%)	16 (37%)
Follow-up <12 months	12 (18%)	12 (18%)	19 (29%)	23 (35%)
Follow-up ≥12 months	1 (6%)	5 (28%)	3 (17%)	9 (50%)

Abbreviations: RCT: randomized controlled trial; c-RCT: cluster randomized controlled trial; OR: odds ratio.

In general, the inclusion of other non-medical professionals seems to be most successful in improving physician adherence to guidelines. Large treatment effects are indicated in 57% of comparisons where non-medical professionals were included in the implementation process, compared to 38% if exclusively physicians were included, and 42% if nurses were included. Furthermore, large treatment effects are more frequently found in trials on primary or secondary prevention (48 or 40% of trials) compared to tertiary prevention (24%) of CVD.

Comparisons of trials investigating the process of implementation with physician adherence as a primary outcome less frequently showed a negative direction of effect (12% vs. 19%) and more frequently a moderate or large effect size with OR>1.2 (71% vs. 58%). Finally, RCTs were more frequently successful with a moderate or large effect size compared to c-RCTs (79 vs. 58%), and studies with longer follow-up periods of at least 12 months more frequently showed a large effect size (50% vs. 35%) compared to shorter trials.

Table 3 summarizes the ratios of odds ratios (ROR) for the influence of all investigated clinical and methodological heterogeneity factors on treatment effect. Between-trial variability and the relative reduction of between-trial variability by single effect modifiers compared to the variability of the original model describe the reduction of statistical heterogeneity.

**Table 3:** Association of different effect modifiers on treatment effect.

Potential effect modifier	Comparisons	ROR; 95% CI	Between-trial variability (τ^2^); relative reduction
No effect modifier			0.1899
Staff	Nurse as receiver vs. others	1.29; 1.05 to 1.60	0.1389; 26.9%
	Other professionals as receiver vs. others	1.62; 1.29 to 2.04	
Level of prevention	Primary prevention vs. others	1.30; 0.98 to 1.71	0.1692; 10.9%
	Secondary prevention vs. others	1.31; 1.09 to 1.57	
Unit of randomization	c-RCT vs. RCT	1.28; 1.03 to 1.60	0.1871; 1.5%
Outcome definition	Adherence as primary vs. secondary outcome	1.38; 1.12 to 1.70	0.1719; 9.5%
Duration of follow-up	Long (>12 months) vs. short follow-up periods	1.38; 1.04 to 1.83	0.1741; 8.3%
Risk of bias	Risk of bias (high or unclear vs. low):		0.1488; 21.6%
	Sequence generation	0.88; 0.77 to 1.27	
	Allocation concealment	0.93; 0.64 to 1.33	
	Blinding	1.11; 0.80 to 1.53	
	Incomplete outcome data addressed	1.04; 0.77 to 1.40	
	Selective outcome reporting	1.58; 0.96 to 2.60	
	Other	0.94; 0.74 to 1.20	

Abbreviation: CI: confidence interval; c-RCT: cluster randomized controlled trial; RCT: randomized controlled trial; ROR: ratio of odds ratios.

These results showed that the treatment effect varied depending on clinical heterogeneity factors. The receiver (i.e., person/profession responsible for implementation) of the implementation seems especially to influence the efficiency of implementation strategies on physician adherence. The inclusion of the receiver of the implementation into the meta-regression reduced between-trial variability (τ^2^) by 27%. Physician adherence was improved by 62% (ROR 1.62; 95% CI 1.29 to 2.04) in trials where other non-medical professionals were included in the process of implementation, and expanding the role of nurses in the curative process was successful in improving physician adherence by 29% (ROR 1.29; 95% CI 1.05 to 1.60).

Inclusion of level of prevention as a clinical effect modifier reduced τ^2^ by 11%. Improvement of physician adherence was most successful in the treatment of patients in primary and secondary prevention of CVD (ROR 1.30; 95% CI 0.98 to 1.71 and 1.31; 95% CI 1.09 to 1.57) compared to patients in tertiary prevention.

However, methodological issues seem to have a smaller influence on the investigated association. We found an association between the definition of the primary endpoint as a quantitative measure of the main objective of a trial and the estimated implementation effect. The inclusion of this effect modifier was able to reduce τ^2^ by 10%. The implementation effect on the primary outcome in process optimization studies with the primary endpoint of physician adherence was increased by 38% (ROR 1.38; 95% CI 1.12 to 1.70) compared to outcome optimization studies where physician adherence was investigated as a secondary or process of care parameter. Moreover, we also found an increased implementation effect in RCTs compared to c-RCTs by 28% (ROR 1.28; 95% CI 1.02 to 1.60) and with longer duration of follow-up (ROR 1.38; 95% CI 1.03 to 1.83), with only small reductions of τ^2^ by 2 and 8%, respectively.

Inclusion of six risk of bias domains reduced between-trial variability by 22%, but no single component was associated with a significant overestimation of implementation effect. We found a tendency to an overestimation of implementation effects in trials with potential bias by selective reporting of the primary outcome by 58% (ROR 1.58; 95% CI 0.96 to 2.60).

## Discussion

This study is based on an analysis of 75 trials and on eight classes of implementation strategies to improve physician's guideline adherence. It investigates the influence of six possible effect modifiers on estimated implementation effects and remaining statistical heterogeneity. These influences are quantified by RORs and the relative change of between trial variability. Our investigations revealed a substantial reduction of statistical heterogeneity explained by five of the investigated effect modifiers.

We found that clinical effect modifiers such as the cooperation of physicians with non-medical health professionals, the setting of the primary care of patients in early prevention of CVD, and the duration of implementation were especially associated with the improvement of physicians' adherence to guidelines. We have found a considerable reduction of statistical heterogeneity by these factors of 27, 11, and 8%, respectively. Furthermore, the inclusion of methodological effect modifiers as different sources of bias or the definition of the primary endpoint was able to reduce statistical heterogeneity by 22 and 8%, respectively.

A considerable amount of statistical heterogeneity is explained by organizational structures in the primary care practices. Improvement of adherence could be achieved if physicians accepted support from non-medical health professionals such as pharmacists, health workers, qualified nurses, or nurse practitioners in improving their professional and organizational performance. Such cooperation could take place within team or (smaller) teamlet structures in single practices or networks of care [36]. Teamwork among different professions and/or professionals aimed at implementing guidelines explains the different estimated treatment effects on the adherence of primary care physicians. Our results are in line with the conclusions of Grol and Grimshaw (2003) [37] and Unverzagt et al. (2013) [6] which suggest that the whole primary care team (or network) is important for the implementation success.

We have further showed that different levels of prevention may cause heterogeneous treatment effects with greater improvements in physician adherence in the primary care of patients in the early prevention of CVD. Guidelines are more difficult to implement in tertiary prevention, where patients frequently suffer from complex co-morbidities and guidance on interventions and information on risks of specific interventions is missed [38]. This observation reflects the problem how far results from RCT can be generalized due to selection in patients included in RCTs and narrow inclusion/exclusion criteria [39]. Especially patients with complex comorbidities should be well represented in RCTs and guidelines should provide guidance for the often complex need of these patients including information on risks of specific interventions [38].

Moreover, our explorations revealed artificial sources of variation resulting from the inclusion of two different types of study with different main aims, although both types reported physician adherence. Physician adherence summarizes the degree of conformity between knowledge, cognition, and action in a primary physician center with evidence-based recommendations from guidelines on different aspects of quality of care [40].

The primary aim of the first type of study is to improve the care process by implementing guidelines and the primary aim of the second type is to improve the health outcomes of patients. A “change” to a more evidence-based treatment aimed at improving health outcomes is a stepwise complex process in which several individual and organizational barriers have to be removed and intermediate outcomes (such as adherence) and final outcomes (such as health outcomes) may be improved. First, different strategies should be used to implement evidence-based guidelines in the care process and to enhance the adherence of physicians. Secondly, patients must be adherent to evidence-based recommendations, and finally, the health outcomes for patients might change. The benefit they receive from guideline-oriented treatment by reduced hospital admissions and prolonged survival has been shown in several studies [41]–[43]. In trials designed to improve the implementation of guidelines, physician adherence was regularly chosen as a primary endpoint and measured by multiple indicators, which were summarized to adherence scores or described by different frequencies.

The second type of study concentrates on the improvement of health outcomes of patients and describes physician adherence as a step towards improving mortality, morbidity, health-related quality of life, or surrogate parameters such as the achievement of targets for blood-pressure, cholesterol concentrations, physical activity, body mass index, smoking cessation or reduced smoking, or the reduction of cardiovascular risk score. Some statistical heterogeneity of treatment effects can be explained by these different types of studies.

In addition, we stated an increased treatment effect in studies with longer follow-up periods where implementation strategies were used over a longer period with a potentially better chance to improve providers knowledge, to integrate guidelines recommendation into organizational structures and processes and to overcome as well as negative staff attitudes and beliefs and time and resource constraints in primary care centers.

Furthermore, we identified an influence of risk of bias on the variability of treatment effect and were able to reduce statistical heterogeneity, but we were not able to identify one single source of bias causing biased treatment effects. However, different additional factors show divergent influences on the process of implementation and health outcomes and may influence both the process of implementation and the efficacy of the recommended intervention for the patient. These factors include concerns about the quality of guidelines such as the quality of evidence on which they are based, lack of agreement, differences in strength of recommendations, practicability of guidelines and recommended interventions, and the benefit/harm ratio of the intervention [44], [45]. Finally, financial constraints and organizational structures (e.g., health systems) may modify the process of care [46].

### Strengths and limitations

We are aware that our research may have some limitations. The conclusions of our systematic review [6] were limited by a large amount of imprecision and inconsistency of reported results [24], [47], [48]. Unexplained heterogeneity was caused by different methodological and clinical effect modifiers and some of them were investigated in this study. Other, not investigated potential effect modifiers are differences in health care systems, stability, attitudes and resource constraints of health care teams, types of guideline-recommended interventions, patient decisions and treatment. This study contributes an improved insight into the processes and elements of successful change and shows the environment in which implementation strategies work best, but all these ideas are exploratory and should be considered as hypotheses for evaluation in future studies [17]. Developing of precise definitions of effect modifiers for complex definitions of participants, intervention and outcomes was problematic because our priori definitions were influenced from our growing knowledge on existing evidence. To reduce the dependence on the results of data syntheses, we discussed these potential effect modifiers and tried to find the best classifications on the basis of diverse extracted clinical and methodological characteristics before we started data synthesis. Moreover, we are assured that the investigated heterogeneity factors are unlikely to influence the pathway from implementation strategy to an improved physician adherence and patient outcome independently and may reflect associations with other correlated but not investigated factors [25]. Furthermore, it is possible that especially in studies with inclusion of patients in early and late prevention relationships across trials may not be the same as relationships for patients within trials [29].

## Conclusions

We recommend a careful discussion of the pathway from the intervention to the outcomes and pre-definition of potential effect modifiers at an early time point when conducting a systematic review. This seems to be especially important in a review of complex interventions with a broad range of participants, interventions, comparators, and definitions of outcomes. Our work has explained some statistical heterogeneity by clinical and methodological effect modifiers. These effect modifiers cause variability in estimated treatment effects, and taking them into account reduced statistical heterogeneity.

The results of this study provide some evidence that the incorporation of different health professionals in the practice can change professional and organizational performance in a primary care practice. This study properly investigated the role of implementation strategies in the treatment of patients in primary and secondary prevention of CVD and provides some evidence of promising results. However, the implementation of guidelines into tertiary prevention of CVD in general practice requires improved guidance for this patient group. Finally, we propose choosing the process parameter of physician adherence as the primary outcome parameter only in cases where a theoretical model explains the route from the intervention to the anticipated health outcomes for patients. Nevertheless, the health outcomes for patients should always be measured and reported additionally to process parameters (e.g., physician adherence) to ensure the association between the process parameter and improved health outcomes and to improve understanding of the pathway from implementation over physician adherence to improved health outcomes.

## Supporting Information

Table S1 **Included studies and comparisons with treatment effect, implementation strategies in the intervention group, risk of bias and effect modifiers.** Abbreviations: lnOR: logarithmic Odds Ratio, SElnOR: standard error of logarithmic Odds Ratio.
(XLSX)
